# Magnesium oxide nanoparticles modulate phase separation to form trabecular-structured cryogels for bone defect repair

**DOI:** 10.1016/j.mtbio.2025.101631

**Published:** 2025-03-05

**Authors:** Botao Liu, Mingming Hao, Jianping Chen, Xiaodong Hu, Jiaqi Zhong, Yujiong Chen, Han Yu, Hangbin Weng, Zhewei Zhang, Tianyu Du, Zhaoxiang Peng

**Affiliations:** a Affiliated Li Huili Hospital, Ningbo University, Ningbo, 315040, PR China; b Health Science Center, Ningbo University, Ningbo, 315211, PR China; c I-lab, Suzhou Institute of Nano-Tech & Nano-Bionics (SINANO), Chinese Academy of Sciences (CAS), Suzhou, Jiangsu, 215123, PR China

**Keywords:** Magnesium oxide, Phase separation, Cryogel, Biomimetic material, Bone regeneration

## Abstract

Critical-sized bone defects pose notable therapeutic challenges and often require extensive bone grafts for effective intervention, leading to a substantial medical burden. The scarcity of autologous bone and the complex architecture of trabecular bone necessitate the development of cost-effective biomimetic graft materials. In this study, we developed a MgO nanoparticle-incorporated hydrogel scaffold (P-G-C-MgO2) using a freeze-induced phase separation approach. The scaffold achieved a porous structure with 56.48 ± 7.062 % porosity and an average pore size of 565.7 ± 53.62 μm, closely mimicking natural trabecular bone. It demonstrated exceptional mechanical stability during degradation and consistently released bioactive components, including Mg^2+^, type I collagen, and gelatin. These features facilitated early cell recruitment and osteogenic differentiation. In a distal femoral bone defect model, P-G-C-MgO2 exhibited excellent osseointegration and significantly enhanced new bone regeneration. This biomimetic design offers a promising solution for bone defect repair. Moreover, it established a novel phase-separation-based strategy for fabricating porous hydrogel scaffolds.

## Graphical abstract

Magnesium oxide nanoparticles modulate the phase separation of gelatin, type I collagen, and PVA during the freezing process, creating a biomimetic trabecular porous hydrogel structure. As the hydrogel degrades, it releases magnesium ions and bioactive components, which effectively recruit stem cells to the defect site. The cells colonize the porous matrix and are then induced to differentiate osteogenically, achieving efficient repair of bone defects.

**Figure:**
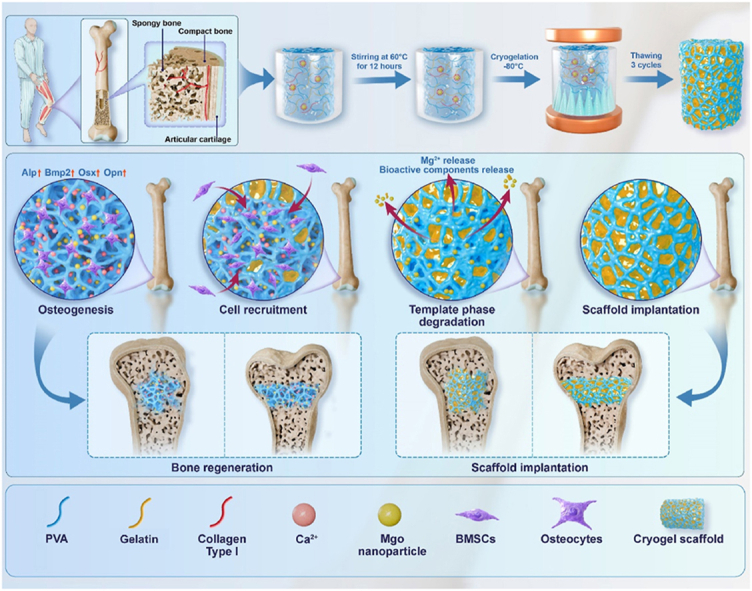

## 1 Introduction

**Scheme 1:**
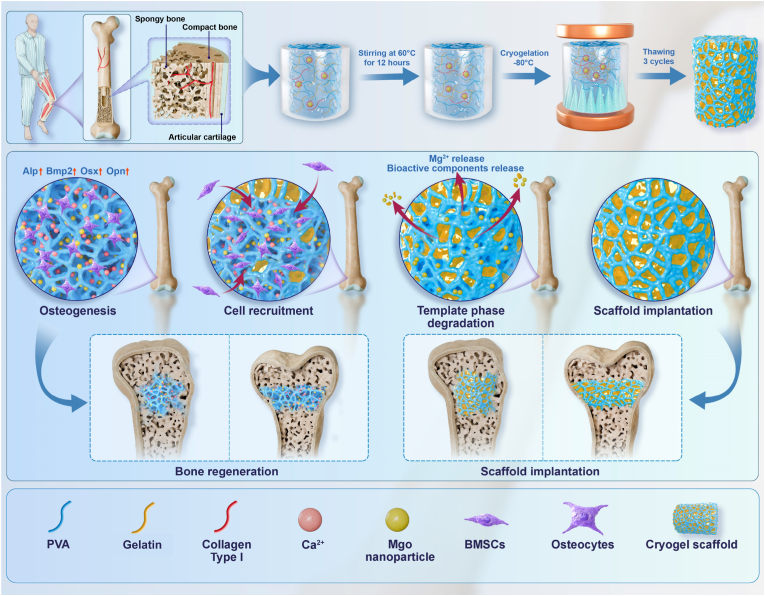
Fabrication process and biological function of the P-G-C-MgO2 hydrogel scaffolds.

Critical bone defects caused by trauma, tumor resection, or inflammation represent a notable challenge in orthopedic clinical practice [[1], [2], [3]]. Traditional artificial bone materials facilitate bone repair in critical bone defects by achieving volumetric filling and structural reconstruction [4]. However, the absence of shape memory capabilities in these implants makes them susceptible to irreversible damage under dynamic stress, compromising structural support [5,6]. Additionally, traditional materials lack bionic porosity, frequently causing inadequate bone-implant integration and reduced repair efficacy [[7], [8], [9]]. Consequently, the development of novel bone-filling materials must not only improve dynamic adaptability but also emphasize the role of biomimetic porosity in enhancing bone healing (see Scheme 1).

Cryogels have advantages, such as ease of fabrication, tunable mechanical properties, and an intrinsic porous structure. Specifically, polyvinyl alcohol (PVA) can spontaneously form hydrogen bonds between molecular chains using freeze-casting technology, creating strong physical cross-links and rendering it highly suitable for orthopedic applications [[10], [11], [12], [13]]. Previous studies have shown that repeated freeze-thaw cycles further restrict PVA chain migration, producing a hydrogel scaffold characterized by shape memory, high compressive strength, and enhanced energy absorption [14]. Nevertheless, with regard to pore structure, the pore size of PVA cryogels is frequently confined to below 100 μm due to ice crystal formation limits, making it challenging to construct trabecular bone-mimicking porous hydrogels from pure PVA [[15], [16], [17], [18]].

Phase separation technology, wherein two or more immiscible gel precursor solutions are mixed, can induce biphasic polymer structures through the precise modulation of factors, such as temperature, reagent ratios, and pH. This approach is increasingly recognized as an effective method for obtaining porous structures [[19], [20], [21]]. With the tailored design of cryogel precursor solutions, when reaching the gel-sol transition temperature during freezing, the second phase preferentially precipitates near the nucleation sites. This second phase acts as a template, extruding the first phase into the interstices of the template [22,23]. Achieving these effects requires precise modulation of freezing parameters and careful selection of nucleation inducers [24,25]. Ma et al. [26] controlled the porosity of PVA-gelatin cryogels by incorporating nano-hydroxyapatite, achieving a target porosity of 76.3 %, closely resembling physiological bone and potentially enhancing metabolic exchange. However, in addition to achieving high porosity, biomimetic trabecular structures require sufficient pore size to support endogenous bone tissue growth. Additional research on phase-separated cryogels is required to develop scaffolds with high porosity, physiological pore sizes, and enhanced connectivity [27,28]. As biomaterials, the concentration and release profiles of nucleation inducers must be studied further to determine their effects on biosafety and bone healing.

Magnesium ions, the fourth most abundant cations in the human body, are readily metabolized and possess a broad safety margin [29,30]. Studies have demonstrated that magnesium ions enhance the bioresponsiveness of materials, facilitating cellular recruitment, adhesion, and proliferation. Furthermore, magnesium ions play a crucial role in initiating bone cell biomineralization, with their presence optimizing the osteogenic induction of mesenchymal stem cells and thereby accelerating bone repair [31,32]. Magnesium oxide nanoparticles, magnesium ion derivatives, gradually release magnesium ions within the body without inducing rapid hydrogen gas release or significant pH changes [33,34]. Moreover, magnesium oxide nanoparticles have a high surface area and positive charge, enabling them to attract negatively charged gelatin molecules. This electrostatic interaction provides nucleation sites, where the magnesium oxide nanoparticles function as heterogeneous nucleating agents to induce uniform second-phase precipitation at controlled temperatures.

In this study, we used a magnesium oxide nanoparticle-induced nucleation strategy with temperature-controlled phase separation by blending PVA, gelatin, type I collagen, and magnesium oxide nanoparticles. During freezing, the porogenic template and polymer phases formed sequentially, creating interconnected porous trabecular-structured hydrogel scaffolds. Compared with traditionally layered hydrogels, dual-phase hydrogels synthesized via this simple, cost-efficient phase separation technique not only exhibited exceptional biomechanical properties but also allowed for flexible control of structure and physicochemical properties. Furthermore, the hydrogels exhibited outstanding bioactivity *in vitro* by releasing bioactive components during degradation, effectively promoting cell recruitment and osteogenic differentiation. Additionally, they demonstrated remarkable efficacy *in vivo* in repairing critical-sized bone defects.

## 2 Materials and methods

### 2.1 Materials

Polyvinyl alcohol (PVA-210, molecular weight ∼67,000 g/mol), gelatin [from porcine skin, gel strength 260–330 (Bloom) < 10 EU/g endotoxin], and magnesium oxide (MgO) nanoparticles (50–100 nm, 99 %) were procured from Shanghai Aladdin Industrial Corporation Co., Ltd. (Shanghai, China). Collagen (cow calcaneal tendon, Type I) was procured from Shanghai Maclin Biochemical Technology Co. Ltd. (Shanghai, China). Simulated body fluid (SBF; pH 7.4) and phosphate-buffered saline (PBS; pH 7.4) were purchased from Beijing Solarbio Science &Technology Co., Ltd. (Beijing, China). All other materials utilised in this study were of commercial grade and were used as received without further modification.

### 2.2 Synthesis of P, P-G, P-G-C, and P-G-C-MgO hydrogel scaffolds

The preparation of hydrogels via freeze-casting and phase separation involves several steps. Initially, 15 g of PVA was dissolved in PBS under continuous mechanical stirring at 80 °C for 6 h, resulting in a 15 % (w/v) PVA solution, designated as Solution A. The solution was allowed to cool to room temperature for subsequent use. Concurrently, 15 g of gelatin and 4 g of collagen were separately dissolved in PBS and stirred at 60 °C for 6 h to form a 15 % (w/v) gelatin solution (Solution B) and a 4 % (w/v) collagen solution (Solution C). Both solutions were cooled to room temperature for later use. Next, equal volumes of Solution A (100 mL) and PBS (100 mL) were mixed and stirred at 60 °C for 12 h to prepare the P solution, which contained 7.5 % (w/v) PVA. Similarly, Solutions A and B were mixed in specific proportions, diluted with PBS, and stirred for 12 h to obtain a P-G solution containing 7.5 % (w/v) PVA and 5 % (w/v) gelatin. To prepare the P-G-C solution, Solutions A, B, and C were mixed in specific proportions, diluted with PBS, and stirred at 60 °C for 12 h, resulting in a mixture containing 7.5 % (w/v) PVA, 5 % (w/v) gelatin, and 1 % (w/v) collagen. To prepare P-G-C-MgO*n* (where *n* denotes the different w/v percentages of MgO), varying amounts of MgO were added to the mixture of solutions A, B, and C, followed by dilution with PBS and stirring at 60 °C for 12 h. This process yielded mixtures containing 7.5 % (w/v) PVA, 5 % (w/v) gelatin, 1 % (w/v) collagen, and 0.1, 0.5, 1, 2, or 3 % (w/v) MgO. Subsequently, these solutions were poured into Teflon moulds and frozen at −80 °C for 24 h, followed by natural thawing at room temperature for 12 h. This freeze-thaw cycle was repeated thrice to fabricate hydrogel scaffolds.

### 2.3 Characterization of the hydrogel scaffolds

The micromorphologies of the hydrogel scaffolds were observed using scanning electron microscopy (SEM) with an energy-dispersive spectrometer (SEM-EDS, S-4800; Hitachi, Tokyo, Japan) at an accelerating voltage of 15 kV. The surface functional groups of the samples were detected using Fourier transform infrared (FTIR) spectroscopy (Nicolet iN10; Thermo Fisher Scientific, Waltham, MA, USA) and an X-ray Diffraction System (Rigaku SmartLab SE; Rigaku Corporation, Tokyo, Japan). The zeta potentials of the samples were measured at 25 °C (Malvern Zetasizer Nano ZS90; Malvern Panalytical, Malvern, UK).

### 2.4 Degradation behavior of the hydrogel scaffolds

The hydrogel scaffolds were prepared by cryogelation using 10 mL of a homogeneous reaction solution and were subsequently used as samples for the degradation experiments. The hydrogel scaffolds were incubated at 37 °C in a PBS solution containing 2 μg/mL collagenase II. The solutions were replaced periodically. Thereafter, the scaffolds were removed from the solution, rinsed with deionized water at the indicated times (1, 3, 7, 14, 28, and 56 d), dried with filter paper, and weighed. The degradation ratios (%) of the scaffolds were calculated as follows:

(1) $$\text{Degradation}\;\text{ratio}\;\left(\%\right)=\frac{W1-W2}{W1}\times 100\%$$

where *W2* and *W1* are the weights of the remaining and initial scaffolds, respectively, at different time points. The *in vitro* Mg^2+^ release behavior of the P-G-C-Mg hydrogel scaffolds and the Mg^2+^ concentrations in the liquid extracts of the samples were measured using inductively coupled plasma optical emission spectrometry (ICP-OES; Thermo Fisher Scientific) at the indicated times. The change in the pH of the liquid extracts was measured using a pH metre (Mettler Toledo, Greifensee, Switzerland).

### *2.5* *In vitro* mineralization study

The hydrogel scaffolds were immersed in SBF for the *in vitro* mineralization study, as described previously [35,36]. Specifically, the samples were placed on a shaker and incubated in SBF at 37 °C for two weeks, with the SBF refreshed every 2 d. After mineralization, the scaffolds were freeze-dried and analyzed using SEM. The remaining unfrozen samples were carefully blotted to remove surface moisture and were prepared for subsequent experiments.

### 2.6 Pore structure analysis

After *in vitro* mineralization, the hydrogel scaffolds were scanned using a micro-CT system (VENUS001; PINGSENG Healthcare Inc., Shanghai, China). Scanning was performed under a tube voltage of 90 kV and a tube current of 0.08 mA, with a continuous scanning acquisition mode employed to collect the data. The scanning data were then reconstructed using the FDK algorithm in Avatar software (Avatar Software, LLC, Franklin, TN, USA), yielding three-dimensional reconstructed images of the hydrogel scaffolds. The entire hydrogel scaffold was selected as the region of interest (ROI) for further analysis. A standardized threshold (>-350) was applied to define the hydrogel scaffold, with the remaining region classified as the pore space between the scaffolds. The pore porosity, pore size, specific surface area, and connectivity were analyzed and quantified within the selected ROI.

### 2.7 Mechanical properties and shape-memory effect of the hydrogel scaffolds

An Instron Universal Testing Machine (Instron, Norwood, MA, USA) was used to evaluate the compressive mechanical properties of the hydrogel scaffolds (10 mm high and 20 mm wide). Commencing with an initial distance of 15 mm, a loading rate of 2 mm/min was applied (the total test distance was 60 or 40 % of the sample height), with the procedure replicated across three parallel samples for each group. The compression modulus was calculated based on the slope of the stress-strain curve at a strain of 10 %. Seventy, twenty-four, and one hundred fifty cycles of compression testing for the scaffolds (P-G-C-MgO2, P-G-C-MgO2 after mineralization, P-G-C-MgO2 degradation in PBS solution for four weeks) were conducted at a strain rate of 2 mm/min with a maximum strain of 60 or 40 %. The storage (G′) and loss (G″) moduli of the scaffolds were assessed using a HAAKE MARS 60 rheometer (Thermo Fisher Scientific) at an oscillation frequency of 0.1–10 Hz and a strain amplitude of 2 %.

### 2.8 Preparation of extract solutions

Each group of hydrogels was prepared under sterile conditions into cylindrical shapes with a diameter of 2 cm and a height of 1 cm following the described protocol. In accordance with ISO 10993-12 guidelines, the hydrogels were immersed in DMEM at a concentration of 0.1 g/mL and incubated at 37 °C with shaking at 200 rpm for 24 h to obtain extract solutions. Subsequently, the extract solutions were supplemented with 10 % FBS (Corning, USA), 200 U/mL penicillin (Beyotime, China), and 200 μg/mL streptomycin (Beyotime, China) to facilitate the next stage of cell experiments.

### 2.9 Biocompatibility assay

Cells were seeded at a density of 5 × 10^3^/mL in 96-well plates and cultured using the extract solutions from each hydrogel group. CCK-8 assays were performed on days 1, 2, and 3 to evaluate cytotoxicity. For live/dead staining assays, the cells were seeded in 12-well plates and incubated in complete medium for 24 h. The medium was then replaced with the extract solutions at designated time points and the cells were stained using a Calcein AM/PI kit (Beyotime) for 30 min, followed by observation under a fluorescence microscope (DMi8; Leica Microsystems, Wetzlar, Germany) to assess cell viability. For the hemolysis assays, fresh rat blood anticoagulated using ethylenediaminetetraacetic acid (EDTA) was centrifuged at 2000 rpm for 5 min, followed by PBS washes to prepare a red blood cell suspension at a density of 2 × 10^8^ cells/mL. Next, 900 μL of the red blood cell suspension was mixed with 100 μL hydrogel extract, PBS, or 1 % Triton-X-100 and incubated at 37 °C for 60 min. Following centrifugation at 2000 rpm for 5 min, the supernatant was collected, and the absorbance at 545 nm was measured for the hydrogel groups (At), PBS (Apc), and Triton-X-100 (Anc). The hemolysis ratio (%) was calculated as follows:

(2) $$\text{Hemolysis}\;\text{ratio}\;\left(\%\right)=\frac{\left(At-Apc\right)}{\left(Anc-Apc\right)}\times 100\%$$

For adhesion assays, rat bone marrow-derived mesenchymal stem cells (rBMSCs) were cultured with the extract solutions for 1 d, fixed with 4 % paraformaldehyde, and permeabilized with 0.1 % Triton-X-100 for 30 min. Thereafter, the cells were stained with phalloidin-FITC and DAPI, and adhesion patterns were observed using fluorescence microscopy.

### 2.10 Cell migration and chemotaxis

The rBMSCs were seeded at a density of 4 × 10^5^ cells/mL in 12-well plates. When the cells reached 80 % confluence, a cell-free scratch was created along the surface of the plate using a pipette tip. Residual debris was removed by washing with PBS, and the medium was replaced with extract solutions containing 2 % FBS. Wound closure was imaged at 0, 12, and 24 h using a bright-field microscope (DMi8; Leica Microsystems). Wound edges were marked using Photoshop software (Adobe Inc., San Jose, CA, USA) and wound area measurements were quantified using ImageJ software (National Institutes of Health, Bethesda, MD, USA) for statistical analysis. For the chemotaxis assay, rBMSCs were seeded in the upper chamber of a Transwell system, and hydrogel extract solutions from the experimental groups were added to the lower chamber. After 24 h of co-culture, the cells in the upper chamber were fixed with 4 % paraformaldehyde for 30 min, washed with PBS, and stained with crystal violet for 1 h. Non-migratory cells were removed with a cotton swab and the migrated cells were visualized using bright-field microscopy and quantified using ImageJ software (National Institutes of Health).

### 2.11 Osteogenic differentiation

Cells were seeded at a density of 5 × 10^4^ cells/mL and cultured in extract medium supplemented with 10 mM β-glycerophosphate, 50 μg/mL ascorbic acid, and 10 nM dexamethasone. Alkaline phosphatase (ALP) staining was performed on day 7 post-induction, and Alizarin Red S (ARS) staining was performed on day 21 using the respective staining kits to evaluate ALP activity and calcium nodule formation, respectively. Further, stained samples were observed under a microscope. For immunofluorescence assays, rBMSCs were seeded onto cell slides and cultured for 7 d in extract medium. Thereafter, the cells were fixed with 4 % paraformaldehyde for 15 min, washed thrice with PBS, and permeabilized with 0.5 % Triton-X-100 at room temperature for 20 min. After additional PBS washes, blocking was performed using 5 % bovine serum albumin for 30 min. Next, the cells were incubated overnight at 4 °C with a primary antibody (Osteopontin Polyclonal Antibody 22952-1-AP, Osteocalcin.

Polyclonal Antibody 23418-1-AP; Proteintech China Wuhan Sanying Biotechnology Co., Ltd., Wuhan, China). After washing, the samples were incubated with a CoraLite488-conjugated Goat Anti-Mouse IgG secondary antibody (SA00013-2; Proteintech China Wuhan Sanying Biotechnology Co., Ltd) for 1 h. Sequential staining with F-actin (30 min) and DAPI (5 min) was performed, and the slides were visualized using a fluorescence microscope. Quantitative image analysis was performed using ImageJ software (National Institutes of Health). For qPCR, total RNA was extracted from cells cultured for 7 d using the TRIzol method and reverse-transcribed into cDNA using a commercial reverse transcription kit. The cDNA was then mixed with SYBR Green qPCR master mix and subjected to real-time quantitative PCR using a LightCycler480II system (Roche, Basel, Switzerland). Gene expression data were normalised against the housekeeping gene β-actin and relative expression levels were calculated using the 2^−ΔΔCt^ method. The sequences of primers used in this study are listed in Table S1.

### 2.12 Animal experiment

All surgical procedures were conducted following the ARRIVE guidelines and approved by the Animal Ethics Committee of Ningbo University (approval number: NBU12656). Eight-week-old male Sprague–Dawley (SD) rats weighing approximately 250 g were used to establish a femoral condylar defect repair model. Following previously reported protocols, the hind limbs were sterilised with povidone-iodine after successful anaesthesia [37]. A longitudinal incision was made along the medial skin and subcutaneous tissue, followed by a blunt dissection of the vastus medialis to expose the femoral condyle using a medial approach through the quadriceps. At the apex of the medial femoral condyle, a guide hole was drilled using a Kirschner wire, with the drill bit oriented parallel to the distal femoral articular surface to create a 2.5-mm diameter trans-cortical defect. Upon confirming the satisfactory alignment of the defect channel, bone debris was thoroughly flushed out using physiological saline. The hydrogel scaffold was fully implanted into the defect and the muscle and fascia were meticulously sutured in layers to seal the exposed site. Subsequently, the rats were euthanized four and eight weeks after implantation surgery. Complete femoral specimens were harvested and fixed in 4 % paraformaldehyde solution for 48 h to prepare for downstream experiments. Additionally, serum samples and vital organs, including the heart, liver, spleen, lungs, and kidneys, were collected for biosafety assessment.

### 2.13 Micro-CT analysis

Femoral specimens collected at predefined time points were scanned using a micro-CT system (VENUS001; PINGSENG Healthcare Inc.) following the protocol described above. The acquired datasets were reconstructed using the FDK algorithm in Avatar software to produce detailed three-dimensional (3D) representations of the femoral specimens. A ROI with a diameter of 2.5 mm was delineated along the hydrogel implantation axis in the reconstructed 3D images, with a standardized threshold (>750) applied to identify bone tissue. Bone-related parameters, including newly formed bone volume (BV), bone volume fraction (BV/TV), trabecular number (Tb.N), trabecular thickness (Tb.Th), and trabecular separation (Tb.Sp), were computed and analyzed within a predefined ROI.

### 2.14 Histological analysis

Bone specimens harvested at various time points were decalcified using a 15 % (w/v) EDTA solution, dehydrated in a graded ethanol series, cleared with xylene, and embedded in paraffin. Five-micrometer-thick sections of femoral specimens were processed for hematoxylin and eosin (H&E) and Goldner's trichrome staining. The formation of new bone tissue, hydrogel scaffold degradation, and microstructural details at the bone defect sites were observed using an optical microscope for detailed visualization.

Organ specimens, including the heart, liver, spleen, lungs, and kidneys, were dehydrated and prepared as paraffin blocks following the same procedure. After sectioning, the samples were stained with H&E. Images of organ tissue sections were captured using an optical microscope (DMi8; Leica Microsystems) to evaluate inflammatory cell infiltration and pathological damage to the organs.

### 2.15 Biochemical analysis

Serum samples collected 8 weeks post-surgery were analyzed using an automated biochemical analyzer (AU5800, Beckman, USA) to quantify biomarkers including creatinine (CR), blood urea nitrogen (BUN), aspartate transaminase (AST), alanine transaminase (ALT), alkaline phosphatase (ALP), and serum magnesium (Mg^2+^) to evaluate the impact of hydrogel implantation on renal and hepatic function and magnesium ion accumulation.

### 2.16 Statistical analysis

All experiments were conducted with a minimum of three replicates and the results are presented as mean ± standard deviation. The statistical significance of the differences between groups was assessed using one- or two-way ANOVA, followed by Tukey's post hoc analysis (for more than two groups). *p* < 0.05 was considered to be statistically significant.

## 3 Results and discussion

### 3.1 Preparation and characterization of P-G-C-MgO2 hydrogel scaffolds

**Fig. 1:**
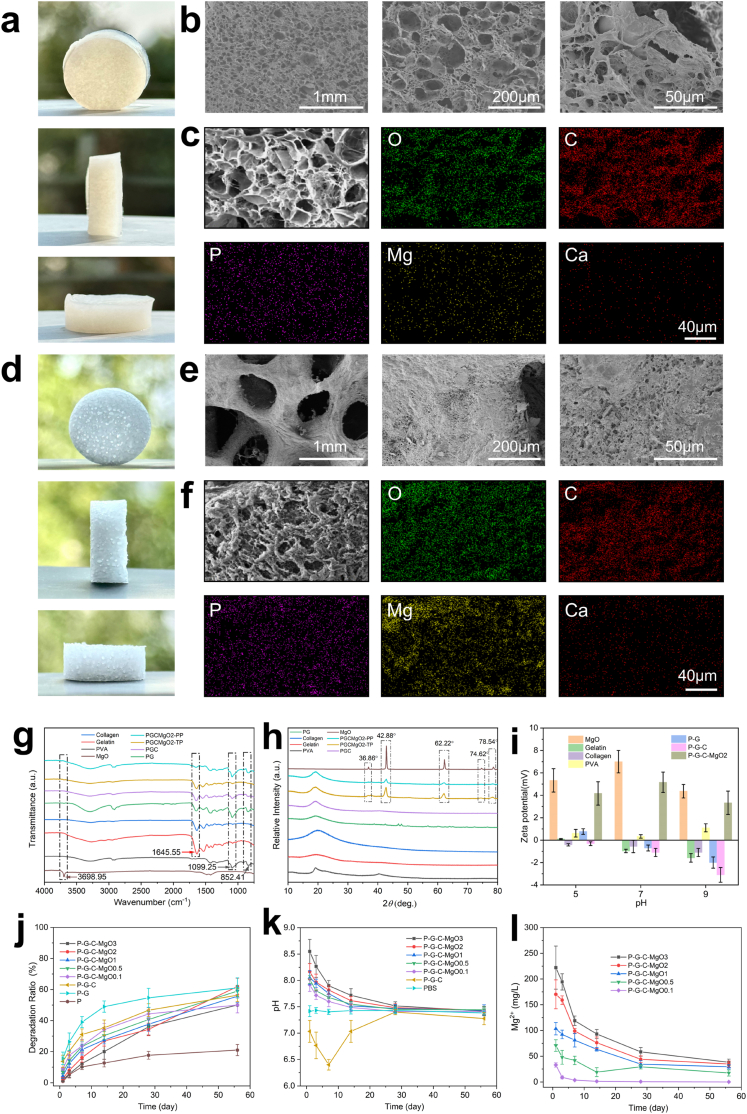
Structural and Characterization of P-G-C-MgO2 hydrogel scaffold. (a–c) Macromorphology, SEM images and EDS mapping of the P-G-C-MgO2 hydrogel scaffold. (d–f) Macromorphology, SEM images and EDS mapping of the P-G-C-MgO2 hydrogel scaffold after *in vitro* mineralization and phase separation. (g–i) FTIR, XRD, and zeta potential of the MgO, PVA, gelatin, collagen, and P-G, P-G-C, and P-G-C-MgO2 hydrogel scaffolds (PGCMgO2-PP, polymeric phase of P-G-C-MgO2; PGCMgO2-TP, template phase of P-G-C-MgO2). (j) Quantitative statistics of the degradation rates of hydrogel scaffolds. (k) pH value monitoring of hydrogel scaffolds during degradation experiments. (l) Mg2+ release from P-G-C-MgO0.1, P-G-C-MgO0.5, P-G-C-MgO1, P-G-C-MgO2 and P-G-C-MgO3 hydrogel scaffolds during degradation experiments.

This study employed freeze-casting and phase-separation techniques to prepare porous P-G-C-MgO*n* (where *n* denotes the different w/v percentages of MgO) hydrogel samples with various concentrations of MgO nanoparticles. Fig. 1a and b and S1a, b show the optical and SEM images of hydrogels with varying MgO contents (P-G-C, P-G-C-MgO0.1, P-G-C-MgO0.5, P-G-C-MgO1, P-G-C-MgO2, and P-G-C-MgO3). An increase in the MgO content from 0 to 0.5 % resulted in a loose, ordered, multiscale porous structure. At higher MgO concentrations (up to 3 %), the pore size decreased and became less ordered. Notably, P-G-C-MgO2 exhibited uniformly distributed pore sizes ranging from 50 to 100 μm, indicating enhanced structural uniformity. Elemental mapping revealed a good distribution of magnesium within the hydrogel matrix. In contrast, calcium and phosphorus were minimally present, consistent with the absence of *in vitro* mineralization (Fig. 1c). Fig. 1d and S2a show optical images of hydrogels with varying MgO contents after 14 d of *in vitro* mineralization. With template phase degradation, the polymer phase skeleton became exposed, forming porous structures and demonstrating that the MgO nanoparticles successfully induced phase separation during freezing. Corresponding SEM images (Fig. 1e and S2b) show that with increased MgO content up to 2 %, P-G-C-MgO2 achieved a trabecular-like multiscale porous structure with pore sizes ranging from 300 to 600 μm and extensive inorganic salt deposition. Mapping analysis indicated that post-mineralization, Ca and P significantly increased, while Mg distribution remained stable, highlighting Mg^2+^ stability and sustained release potential (Fig. 1f).

FTIR and X-ray diffraction (Fig. 1g and h) confirmed the successful incorporation of PVA, gelatin, collagen, and MgO into composite hydrogels and further explored the potential mechanism of phase separation. As shown in Fig. 1g, the P-G-C-MgO2 hydrogel formed two distinct phases during freeze-induced phase separation. The template phase exhibited a characteristic peak at 1645.55 cm^−1^, while the polymeric phase displayed peaks at 1099.25 cm^−1^ and 852.41 cm^−1^, indicating the composite hydrogel separated into gelatin/collagen-dominated template and PVA-rich polymeric phases. In Fig. 1h, characteristic diffraction peaks of MgO (36.86°, 42.88°, 62.22°, 74.62°, 78.54°) were observed in both phases, confirming MgO integration and retention of its crystalline structure. Notably, the template phase exhibited marginally higher MgO peak intensities, suggesting preferential MgO enrichment in this phase, which may modulate the dynamics of phase separation. Fig. 1i illustrates the zeta potential variations of the hydrogel samples and components in buffer solutions with pH values of 5, 7, and 9. MgO displayed a consistently high positive potential across pH levels, whereas PVA, gelatin, and collagen exhibited near-zero or slightly negative potentials. These findings suggest that MgO nanoparticles may regulate phase separation during freezing through electrostatic adsorption, thereby influencing the pore structure. The P-G-C-MgO2 hydrogels exhibited minor zeta potential changes (4–6 mV) across different pH levels, which enhanced their bioactivity and ensured a stable matrix.

The degradation experiment results demonstrated that all hydrogel groups exhibited a rapid degradation rate during the first 20 days, which gradually plateaued thereafter (Fig. 1j). MgO effectively inhibits hydrogel degradation. Thus, P-G-C-MgO2, which had a moderate degradation rate synchronized with the mineralization process *in vivo*, could provide sustained support for bone repair. The pH variations observed during the degradation experiments are shown in Fig. 1k. Initially, MgO hydrolysis caused a pronounced increase in the pH. Over time, the pH gradually declined and stabilized at a mildly alkaline level (approximately 7.4), creating a conducive microenvironment for bone tissue mineralization and regeneration. Fig. 1l illustrates the Mg^2+^ release profiles of hydrogels with varying MgO contents. All samples showed a rapid Mg^2+^ release rate within the first 10 d, followed by stabilization of ion concentration. For P-G-C-MgO2, its Mg^2+^ concentration reached 170.2 ± 28.17 mg/L on day 1, which is within the favorable range for osteogenesis as described in previous studies [38,39]. Higher MgO-content hydrogels sustained prolonged Mg^2+^release, supporting the maintenance of optimal magnesium levels in the biological environment and further promoting bone tissue formation and mineralization.

### 3.2 Characterization of hydrogel scaffold porous structure by Micro-CT

**Fig. 2:**
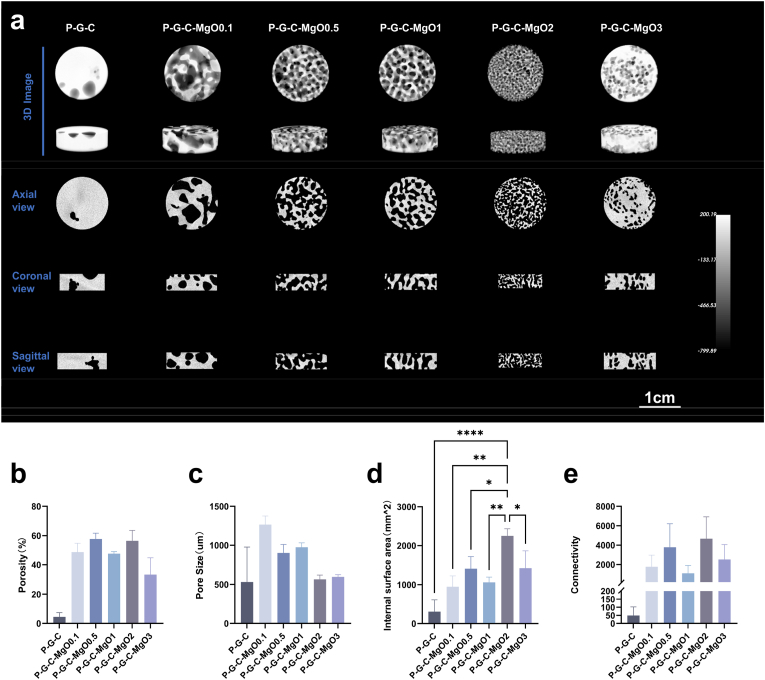
Pore structure analysis of the hydrogel scaffolds. (a) Micro-CT images of P-G-C, P-G-C-MgO0.1, P-G-C-MgO0.5, P-G-C-MgO1, P-G-C-MgO2, and P-G-C-MgO3. (b–e) Porosity, pore size, internal surface area, and connectivity of each group of hydrogel scaffolds. ∗*p* < 0.05, ∗∗*p* < 0.01, ∗∗∗*p* < 0.001, and ∗∗∗∗*p* < 0.0001 by one-way ANOVA with Tukey's post hoc test.

Porous structures play a crucial role in bone defect repair and research has indicated that highly connected multiscale structures with pore sizes close to those of physiological bone, along with high porosity, can significantly promote endogenous bone growth and the effective exchange of metabolites [[40], [41], [42]]. Micro-CT 3D scanning images clearly displayed the overall morphology and internal structural features of the different hydrogel samples. The P-G-C hydrogel structure was relatively dense with less apparent internal porosity; however, as the MgO content increased from P-G-C-MgO0.1 to P-G-C-MgO1, the hydrogel structure became more porous, revealing gradually increasing multiscale porous structures and larger pore sizes. As the MgO nanoparticle content continued to increase, the pore sizes decreased, and the pore structures began to close. P-G-C-MgO2 displayed the most suitable micro/nano multiscale porous structure with open channels (Fig. 2a).

Structural analysis confirmed these conclusions. Hydrogel samples containing MgO nanoparticles exhibited higher porosity, with P-G-C-MgO2, for example, having a porosity of 56.48 ± 7.062 %, which was close to that of physiological bone tissue. However, when the MgO content increased to 3 % (P-G-C-MgO3), the porosity dropped to 33.34 ± 11.56 %, suggesting that excessive MgO nanoparticles might have interfered with phase separation (Fig. 2b). Regarding pore size, as the MgO content increased, hydrogel pore size gradually decreased, from 1265 ± 113.0 μm in P-G-C-MgO0.1 to 565.7 ± 53.62 μm in P-G-C-MgO2. The pore size of P-G-C-MgO3 (597.1 ± 27.19 μm) was similar to that of P-G-C-MgO2 and did not change significantly (Fig. 2c). Furthermore, P-G-C-MgO2, owing to its pore structure being close to that of physiological bone, showed the highest surface area and also possessed a higher connectivity rate with interconnected porous structures (Fig. 2b–e). These structural advantages facilitate endogenous bone growth, vascular ingrowth, and efficient transport of nutrients and metabolites.

### 3.3 Mechanical properties of hydrogel scaffolds

**Fig. 3:**
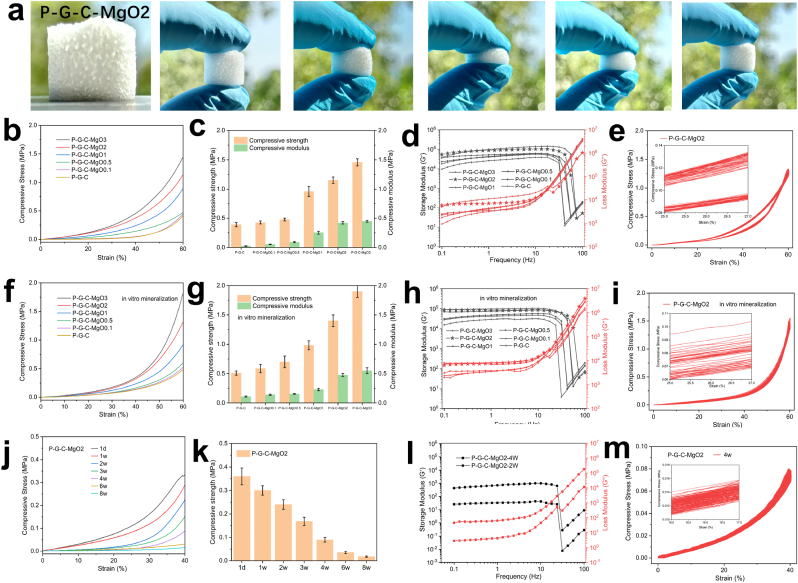
Shape-memory effect and mechanical properties of the hydrogel scaffolds. (a) Rapid shape recovery of the P-G-C-MgO2 hydrogel scaffold. (b–e) Representative compressive stress-strain curves, compressive strength modulus, rheological properties, and stress-strain cyclic curves of each group of hydrogel scaffolds. (f–i) Representative compressive stress-strain curves, compressive strength modulus, rheological properties, and stress-strain cyclic curves of each group of hydrogel scaffolds after *in vitro* mineralization. (j–m) Representative compressive stress-strain curves, compressive strength modulus, rheological properties, and stress-strain cyclic curves of the P-G-C-MgO2 hydrogel scaffolds after degradation for different durations.

The mechanical properties of the hydrogel scaffolds were systematically investigated and correlated with the maintenance of structural integrity post-implantation [43]. For example, Fig. 3a shows that the P-G-C-MgO2 hydrogel sample possesses excellent shape memory characteristics, enabling it to rapidly revert to its original form after deformation. This feature not only reduces the risk of deformation damage during the implantation process but also provides good dynamic matching functionality in the dynamic stress environment of bone defect sites. Fig. 3b and c illustrates the initial compression performance of hydrogels with different MgO contents. The results indicated that the compression stress of the hydrogel sample gradually increased with strain; moreover, a higher MgO content led to greater compressive strength and modulus in the hydrogels. This suggests that incorporating MgO nanoparticles significantly enhanced the compressive performance and structural stability of the hydrogel. The rheological performance results shown in Fig. 3d indicate that within the vibration frequency range of 0.1–30 Hz, the storage modulus (G′) of each hydrogel sample was significantly higher than the loss modulus (G″). This indicated predominantly elastic behavior, which allowed effective energy storage under cyclic external forces. The loss factor (tan δ, Fig. S3a) further reflects the viscoelastic characteristics of the material. All samples exhibited low tan δ values, indicating overall excellent elasticity of the hydrogels. Fig. 3e and S3d show that the P-G-C-MgO2 hydrogel exhibits good strain recovery under cyclic compression, further confirming its high elasticity and deformation-recovery performance.

Fig. 3f and g shows that the compressive performance of the hydrogel samples significantly improved after the mineralization treatment. Samples with higher MgO concentrations, such as P-G-C-MgO2 and P-G-C-MgO3, exhibited higher compressive strengths after mineralization, indicating that *in vitro* mineralization further strengthened hydrogel mechanical performance. The post-mineralization rheological performance displayed in Fig. 3h shows a significant increase in the storage modulus (G′), especially at high MgO concentrations, indicating that the mineralization process enhances the energy storage capacity of the material. The change in tan δ post-mineralization was minimal (Fig. S3b), remaining at low levels, indicating that the high elastic performance of the hydrogel was maintained after mineralization. P-G-C-MgO2 maintained stable strain recovery performance in cyclic compression post-mineralization (Fig. 3i and S3e). This demonstrates that mineralization did not render the hydrogel structure brittle and it still possessed good deformation-recovery characteristics.

Fig. 3j and k depicts the changes in the compressive performance of the P-G-C-MgO2 hydrogel over different degradation times (1–8 weeks). As degradation time increased, the compressive stress of the hydrogel gradually decreased, indicating the impact of the degradation process on mechanical performance. However, the hydrogel maintained a relatively high compressive strength for the first four weeks. G′ gradually decreased over time during degradation (Fig. 3l), reflecting a decline in elastic performance; however, it remained at higher levels for the first 4 weeks. The change in G″ was minimal, indicating that the viscous characteristics of the samples were relatively stable. The tan δ value slightly increased during degradation, however, remained low overall (Fig. S3c), indicating that the P-G-C-MgO2 hydrogel still possessed good elastic performance during degradation. Finally, Fig. 3m and S3f,g demonstrate that after four weeks of degradation, the hydrogel samples maintained a certain compressive strength and deformation capabilities, confirming that they could provide good mechanical support during the mid-term degradation stage.

### *3.4* *In vitro* biocompatibility of hydrogel scaffolds

**Fig. 4:**
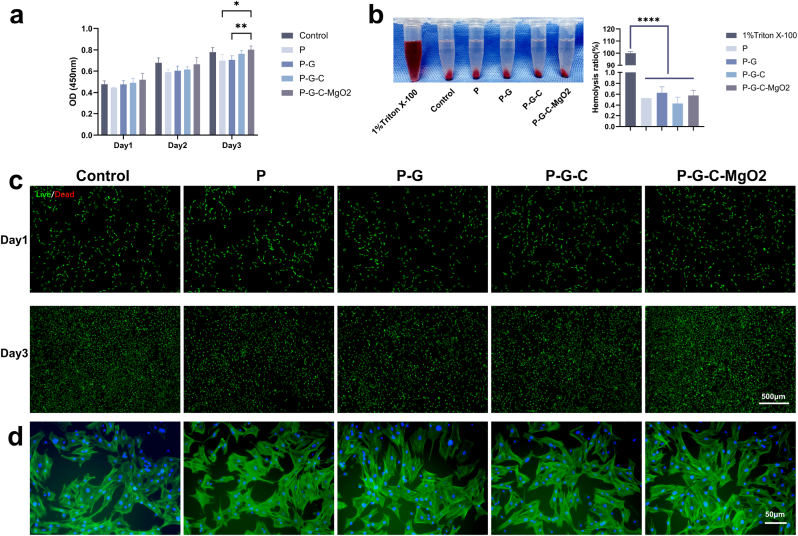
Evaluation of cell compatibility and adhesion of P, P-G, P-G-C and P-G-C-MgO2. (a) CCK-8 test for rBMSCs cultured in the extract of each hydrogel scaffold for 1, 2, and 3 d. (b) Hemolysis test of extracts in each group. (c) Live (green)/dead (red) staining of rBMSCs cultured in the extract of each group. (d) Attachment of rBMSCs observed using fluorescence microscopy. ∗*p* < 0.05, ∗∗*p* < 0.01, ∗∗∗*p* < 0.001, and ∗∗∗∗*p* < 0.0001 by one-way ANOVA with Tukey's post hoc test.

The P-G-C-MgO2 hydrogel demonstrated notable potential for application owing to its advanced structural and mechanical properties. To further confirm the biosafety and bioactivity of the P-G-C-MgO2 hydrogel, subsequent experiments were conducted by sequentially introducing different components to examine their impact on biosafety and effectiveness. rBMSCs, recognized as key effector cells in bone defect repair, were used to assess the *in vitro* biocompatibility and bioactivity of the P-G-C-MgO2 hydrogel components [44]. To this end, cell viability was evaluated using a CCK-8 assay. On day 3, the P-G-C-MgO2 group demonstrated increased optical density values compared to those of the P and P-G groups. This may reflect a synergistic effect between the release of Mg^2+^ and organic components in promoting cell viability. However, on days 1 and 2, cell viability did not significantly differ between the control and the other hydrogel groups, suggesting that each hydrogel component maintained high biosafety (Fig. 4a). Furthermore, the interaction between the degradation products of the hydrogel and the highly localized ionic environment with blood cells serves as a crucial indicator of implant biosafety. Hemolysis assays revealed hemolysis rates below 5 % across all hydrogel samples, signifying no significant haemolytic response or compliance with the blood compatibility standards of the biomaterials (Fig. 4b). Live/dead cell staining images provided direct evidence of cell viability in the extract. On days 1 and 3, high cell viability was observed across all groups without an abnormal distribution of dead red fluorescent cells. Additionally, on day 3, the number of live cells in the P-G-C-MgO2 group markedly exceeded those in the P and P-G groups, with a cell density nearly matching that of the control group (Fig. 4c). This pattern, which aligns with the CCK-8 assay results, underscores the enhanced bioactivity of P-G-C-MgO2, further suggesting that the interplay between Mg^2+^ and organic components may facilitate cell proliferation. In the cell adhesion assay, cells across all groups exhibited normal morphology and developed pseudopodia, enabling early adhesion and spreading in the extract culture environment, thereby supporting subsequent biological processes, such as proliferation, migration, and differentiation (Fig. 4d).

### 3.5 Cell migration and chemotaxis evaluation

**Fig. 5:**
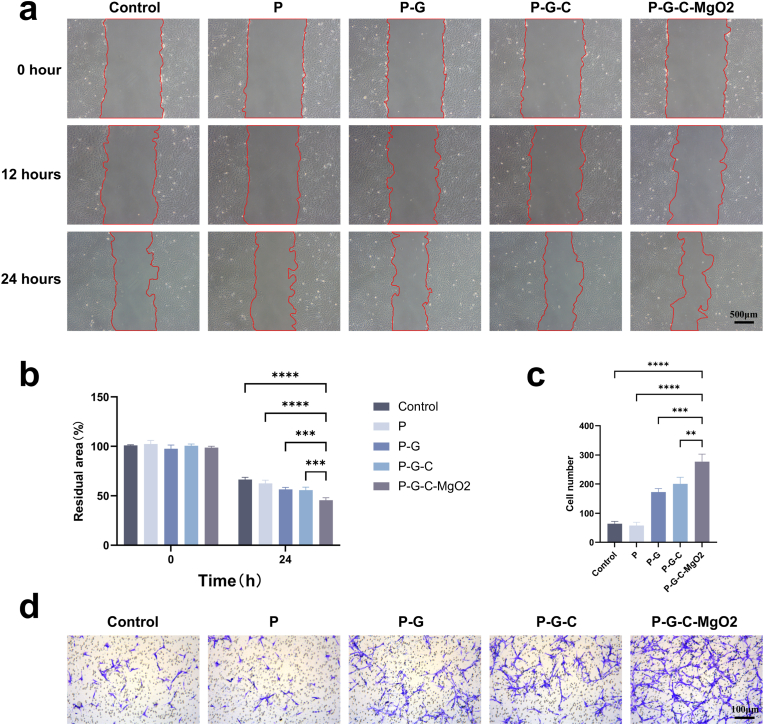
*In vitro* assessment of cell recruitment ability induced by P-G-C-MgO2. (a) Wound healing assay images at 0, 12, and 24 h. (b) Residual area after 24 h of wound healing assay. (c) Cell migration number in cell chemotactic test. (d) Images of crystal violet-stained migrated cells in the cell chemotactic test after 24 h ∗*p* < 0.05, ∗∗*p* < 0.01, ∗∗∗*p* < 0.001, and ∗∗∗∗*p* < 0.0001 by one-way ANOVA with Tukey's post hoc test.

Cell recruitment plays a crucial role in early cellular responses and directly affects the rate of osteogenic healing [45]. To evaluate the recruitment function, wound healing and chemotaxis assays were performed. In the wound-healing assay (Fig. 5a), the cells in each group gradually migrated into the scratch area over time, resulting in a steady reduction in the scratch width. After 24 h, the scratch area in the P-G-C-MgO2 group was reduced to 45.59 ± 2.425 %, showing a significant difference from that of the control (66.35 ± 2.22 %) and P groups (62.47 ± 3.158 %) (Fig. 5b). This increased migration efficiency may be due to the synergistic effects of Mg^2+^ and organic components, such as gelatin and type I collagen, which promote cell movement.

The chemotaxis assay further highlighted the cell recruitment capacity of the hydrogels. In this assay, rBMSCs were seeded in the upper chamber of a Transwell system and hydrogel extracts from each group were placed in the lower chamber. As shown in Fig. 5c and d, the P-G, P-G-C, and P-G-C-MgO2 groups exhibited significantly higher numbers of cells migrating across the Transwell membrane than those in the control and P groups. This suggested that bioactive components, such as gelatin and type I collagen, effectively enhance cell migration. The number of migrating cells in the P-G-C-MgO2 group was also markedly higher than that in the P-G and P-G-C groups, indicating that the synergistic interaction between Mg^2+^ and bioactive components further strengthened cell recruitment capacity.

In summary, the above results suggest that the P-G-C-MgO2 hydrogel can act as a bioactive scaffold during the early implantation stages, efficiently recruiting the surrounding rBMSCs and supporting cellular recruitment, thereby promoting effective bone repair.

### *3.6* *In vitro* osteogenic effect of P-G-C-MgO2 hydrogel scaffolds

**Fig. 6:**
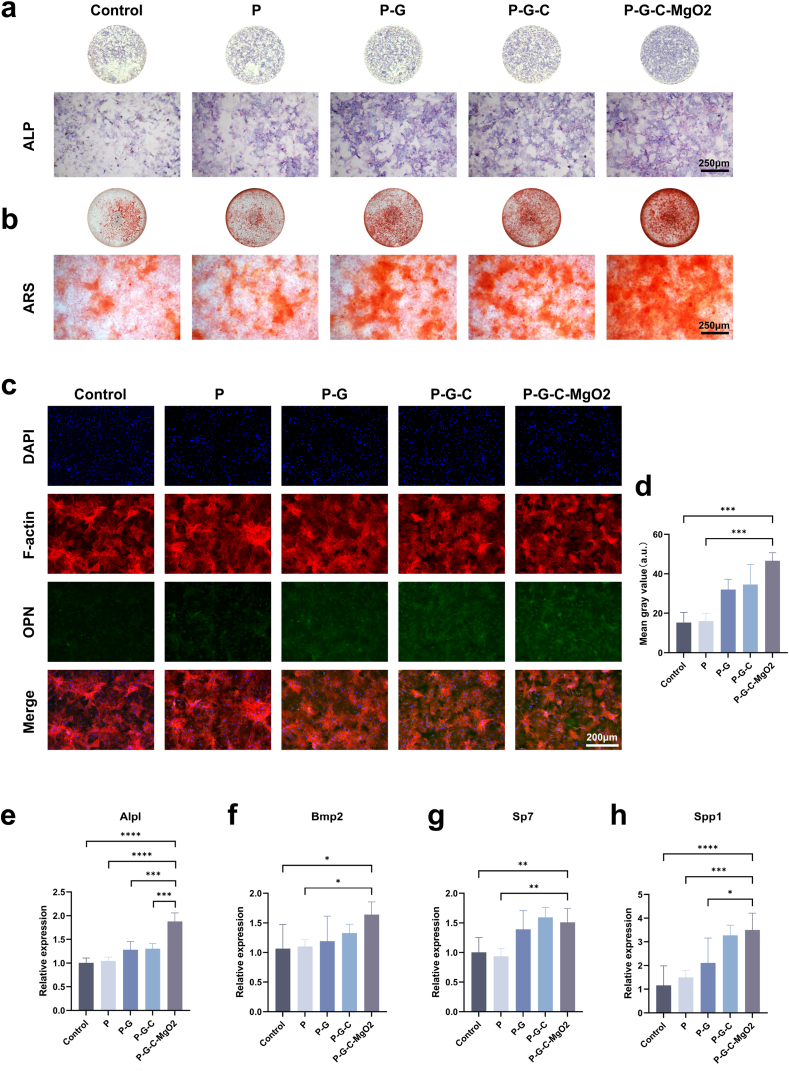
*In vitro* assessment of osteogenic differentiation induced by P-G-C-MgO2. (a)ALP staining of rBMSCs after culturing in conditional medium for 7 d. (b) ARS staining of rBMSCs after culturing in conditional medium for 21 d. (c) Immunofluorescence staining of OPN for rBMSCs in different groups. (d) Quantitative analysis of OPN expression based on immunofluorescence images. (e) RT-qPCR results of *Alpl*, *Bmp2*, *Sp7*, and *Spp1* expression. ∗*p* < 0.05, ∗∗*p* < 0.01, ∗∗∗*p* < 0.001, and ∗∗∗∗*p* < 0.0001 by one-way ANOVA with Tukey's post hoc test.

The osteogenic activity of hydrogel scaffolds is essential for bone repair, particularly after cell recruitment. To illustrate *in vitro* osteogenic activity, rBMSCs were cocultured with conditioned media from each hydrogel scaffold group. ALP staining showed intense purple staining in the P-G, P-G-C, and P-G-C-MgO2 groups, reflecting increased ALP activity in the early stages of osteogenic induction (Fig. 6a). ARS confirmed the presence of more pronounced calcium crystal deposits in the same groups. Among these groups, the P-G-C-MgO2 scaffold exhibited the highest staining intensity, providing further evidence of its capacity to enhance stem cell osteogenic mineralization (Fig. 6b).

Osteopontin (OPN), a protein essential for extracellular matrix mineralization and maturation in bone tissues, plays a crucial role in calcium nodule formation. As shown in Fig. 6c, green fluorescent labelling indicated notably higher OPN expression in the P-G, P-G-C and especially the P-G-C-MgO2 groups. Quantitative analysis revealed a significant increase in OPN expression in the P-G-C-MgO2 group compared with that in the control and P groups, with an upward trend observed across all groups tested (Fig. 6c and d). The same trend was also observed in osteocalcin (OCN) expression, with the P-G-C-MgO2 group exhibiting the highest OCN expression (Figs. S4a and b). These results indicate that the P-G-C-MgO2 hydrogel effectively boosts the synthesis of osteogenic matrix proteins *in vitro*. Additionally, key osteogenic genes, including *Alpl*, *Bmp2*, *Sp7*, and *Spp1*, which are crucial for osteoblast differentiation, showed elevated expression levels that were closely associated with osteoinductive potential. Gene expression analysis revealed marked upregulation in the P-G-C-MgO2 group compared with that in the control and P groups. These findings supported the conclusion that the combined action of Mg^2+^ and organic bioactive components synergistically affected osteoinduction (Fig. 6e–h). This interaction upregulates osteogenesis-related genes, promotes osteogenic protein secretion, and enhances overall osteogenic activity.

### 3.7 Analysis of new bone formation using micro-CT

**Fig. 7:**
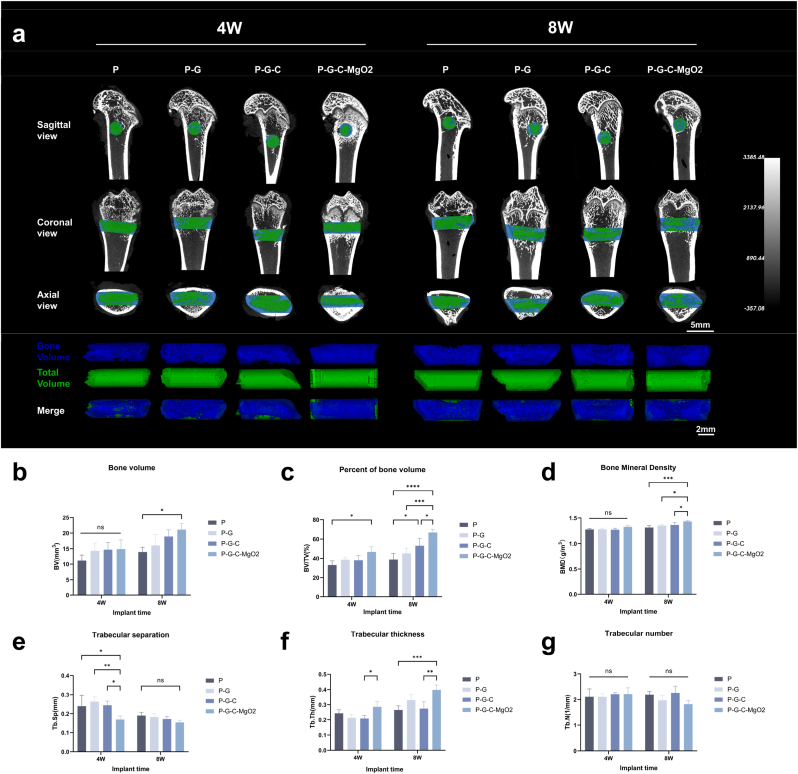
*In vivo* analysis of the new bone formation of P-G-C-MgO2 in the diatal femoral defect in the SD rat using Micro-CT. (a) Micro-CT images of the femur and reconstructed bone tissue images within the defined region of interest (ROI) (blue represents regenerated bone; green represents the defective bone area). (b–g) Quantitative analysis of new bone formation in ROI 4 and 8 weeks post-implantation. (b) BV, (c) BV/TV, (d) BMD, (e) Tb.Sp, (f) Tb.Th, and (g) Tb.N. ∗*p* < 0.05, ∗∗*p* < 0.01, ∗∗∗*p* < 0.001, and ∗∗∗∗*p* < 0.0001 by one-way ANOVA with Tukey's post hoc test.

We established a femoral distal defect model in SD rats and implanted hydrogels in each experimental group to evaluate their efficacy in repairing bone defects. Structural analyses of the harvested femoral specimens at 4 and 8 weeks postoperatively were conducted using micro-CT scans. As shown in Fig. 7a, each hydrogel scaffold group maintained a consistent fixation effectiveness throughout the observation period. By the 4-week mark, only minimal bone deposition was detected within the defective regions, with no notable endogenic bone ingrowth into the scaffold. We hypothesized that this limited early bone formation may be due to the hysteresis required for scaffold degradation to completely expose the porous structure as well as the natural physiological timeline of bone regeneration. However, by the 8-week time point, all groups exhibited notable endogenic bone growth, with the P-G-C-MgO2 group displaying the most substantial bone deposition upon CT imaging. This suggests that once the hydrogel degrades, the porous scaffold structure provides a suitable framework for bone ingrowth, while the bioactive byproducts of degradation probably facilitate early cell recruitment and enhance new bone formation. Quantitative analysis of BV showed no significant differences among the groups at four weeks. However, at eight weeks, the P-G-C-MgO2 group demonstrated a significantly greater BV than that of the P group (Fig. 7b). Furthermore, the BV ratio in the P-G-C-MgO2 group at eight weeks was the highest among all groups, indicating that the exposed porous structure following scaffold degradation provided ample space for bone tissue growth (Fig. 7c). Additionally, the P-G-C-MgO2 group exhibited a higher calcium deposition density per unit volume than that of the other groups. By the 8-week mark, no significant differences were observed among the groups in terms of trabecular separation or number, suggesting that the increase in BV ratio in the P-G-C-MgO2 group may primarily result from increased trabecular thickness (Fig. 7d–g). These findings indicate that the P-G-C-MgO2 hydrogel exhibited superior fixation and bone defect repair capabilities in the *in vivo* implantation model.

### 3.8 Histological and biochemical analysis

**Fig. 8:**
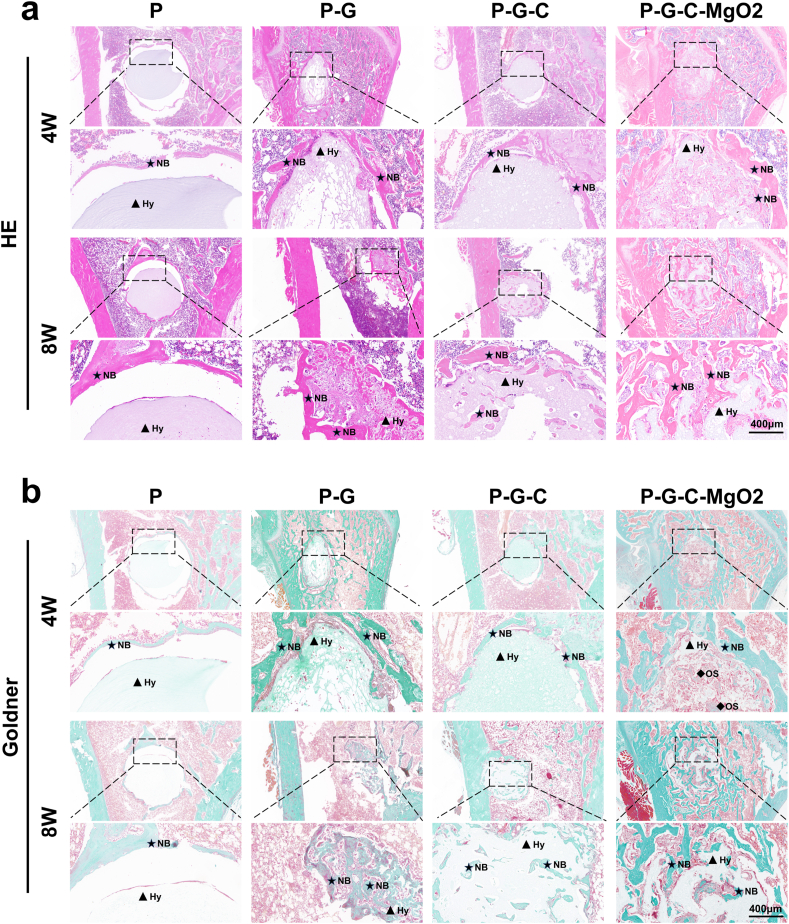
Histological analysis of regenerated bone in the area of rat distal femoral defect observed at 4 and 8 weeks post-implantation. (a) H&E staining of femur specimen after hydrogel implantation. (b) Goldner's trichrome staining of femur specimen after hydrogel implantation. (NB, new bone; Hy, hydrogel; OS, osteoid tissue).

To further evaluate the material structure and efficacy of bone defect repair at the histological level, femoral specimens from each group were subjected to histological sectioning. H&E-stained images revealed that at both 4 and 8 weeks post-implantation, the P group maintained a complete scaffold structure, underscoring the high structural stability of the PVA hydrogel. However, the limited porosity of the P group scaffold resulted in minimal endogenous bone ingrowth, and the bioinert nature of PVA restricted its integration with the surrounding bone tissue. In the P-G group, although gelatin incorporation introduced porosity, the scaffold exhibited poor physical stability, with substantial deformation observed as early as four weeks post-implantation. This reduced mechanical stability may result from delayed gelatin nucleation and precipitation, which likely disrupts PVA chain alignment during freezing. By eight weeks, the P-G hydrogel was completely degraded, resulting in a complete loss of the scaffold structure. Although the degradation products integrated well with the surrounding bone tissue, premature scaffold degradation likely caused a deficit in mechanical support during the critical window for bone regeneration. The P-G-C group demonstrated favorable integration with the surrounding bone tissue post-implantation, likely owing to the release of bioactive components, including gelatin and type I collagen, into the local environment. However, the absence of a sizeable porous structure limited endogenous bone ingrowth, with only minimal bone growth observed at eight weeks. Conversely, the P-G-C-MgO2 group displayed a porous structure and bioactive component release four weeks post-implantation, creating a cell recruitment niche that facilitated substantial stem cell migration into the scaffold. Furthermore, the hydrogel scaffold remained intact and exhibited excellent integration with the surrounding bone tissue. By eight weeks, a significant increase in mineralized bone was observed within the scaffold, indicating that the enhanced osteoinductive properties of the hydrogel promoted the osteogenic differentiation of recruited stem cells, accelerating endogenous bone formation (Fig. 8a).

Goldner staining showed similar results (Fig. 8b), with green-stained mineralized bone closely integrated with the P-G, P-G-C, and P-G-C-MgO2 scaffolds and surrounding tissue. However, only the P-G-C-MgO2 group exhibited extensive red-stained regions, suggesting substantial stem cell recruitment within the porous structure of the scaffold. At eight weeks, the red-stained osteoid tissue in the P-G-C-MgO2 pores had matured to green-stained mineralized bone, indicating denser formation and tighter integration with the hydrogel scaffold compared with those in the other groups.

Regarding *in vivo* biocompatibility, histological examination was performed on the heart, liver, spleen, lung, and kidney tissues harvested from rats eight weeks post-implantation. The results showed that all groups maintained intact tissue architecture comparable to that of normal rats. No signs of inflammatory cell infiltration or pathological damage were observed (Fig. S5a). Haematological indices, including liver and kidney function markers and serum magnesium concentrations, were also analyzed. The results further validated the safety of the hydrogel scaffold *in vivo*, particularly in terms of organ function and ion accumulation. As shown in Fig. S5b, no significant variations in AST, ALT, CR, BUN, ALP, or Mg^2+^ levels were detected across the groups. These findings suggest that the P-G-C-MgO2 hydrogel scaffold and its constituent materials exhibited a high degree of *in vivo* biosafety.

## 4 Conclusion

In this study, we employed MgO nanoparticle-modulated phase separation and freeze-casting to fabricate cryogels with an interconnected porous architecture. The P-G-C-MgO2 hydrogel with a MgO weight percentage of 2 % developed an internal architecture that closely resembled physiological trabecular bone structure. Furthermore, it exhibited remarkable compressive strength and storage modulus both before and after *in vitro* mineralization. In addition, the P-G-C-MgO2 hydrogel displayed exceptional biosafety both *in vitro* and *in vivo*. Compared with pure PVA hydrogels, the integration of bioactive components (gelatin, type I collagen, and MgO nanoparticles) significantly enhanced rBMSC recruitment and osteogenic induction activity *in vitro*. In the *in vivo* study, this scaffold acted as a niche for cell recruitment, demonstrating notable bone defect repair capability. Consequently, owing to its cost-effective preparation, biomimetic trabecular structure, superior mechanical properties, and enhanced bioactivity, the P-G-C-MgO2 hydrogel demonstrates substantial promise as a scaffold for bone tissue engineering in bone defect repair applications.

## CRediT authorship contribution statement

**Botao Liu:** Writing – original draft, Methodology, Investigation, Data curation, Conceptualization. **Mingming Hao:** Writing – original draft, Methodology, Investigation, Data curation, Conceptualization. **Jianping Chen:** Methodology, Data curation. **Xiaodong Hu:** Methodology, Data curation. **Jiaqi Zhong:** Methodology, Data curation. **Yujiong Chen:** Methodology, Conceptualization. **Han Yu:** Supervision, Resources, Funding acquisition. **Hangbin Weng:** Methodology, Conceptualization. **Zhewei Zhang:** Methodology, Conceptualization. **Tianyu Du:** Supervision, Resources, Funding acquisition. **Zhaoxiang Peng:** Writing – review & editing, Supervision, Resources, Project administration, Funding acquisition, Conceptualization.

## Declaration of competing interest

The authors declare that they have no known competing financial interests or personal relationships that could have appeared to influence the work reported in this paper.

## Data Availability

Data will be made available on request.
