# Supplementary material for: Magnesium oxide nanoparticles modulate phase separation to form trabecular-structured cryogels for bone defect repair
Source: Mater Today Bio. 2025 Mar 5;31:101631. doi: 10.1016/j.mtbio.2025.101631 (PMC11929891; doi:10.1016/j.mtbio.2025.101631)
Supplement: Multimedia component 1 [file mmc1.docx]

**Supporting Information**

**
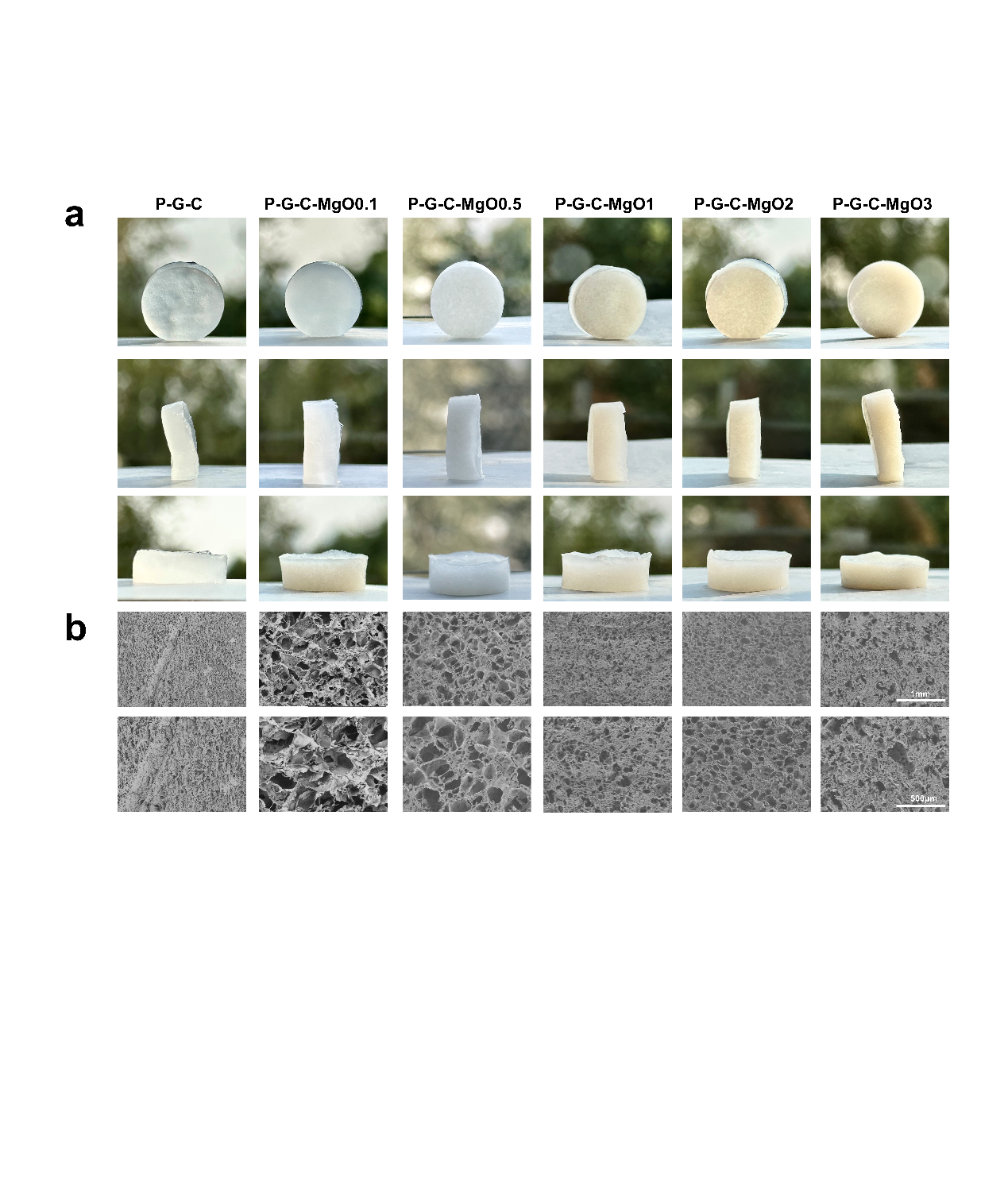
**

**Fig.S1.** **Optical photographs and SEM images of hydrogel scaffolds prior to *in vitro* mineralization**

**
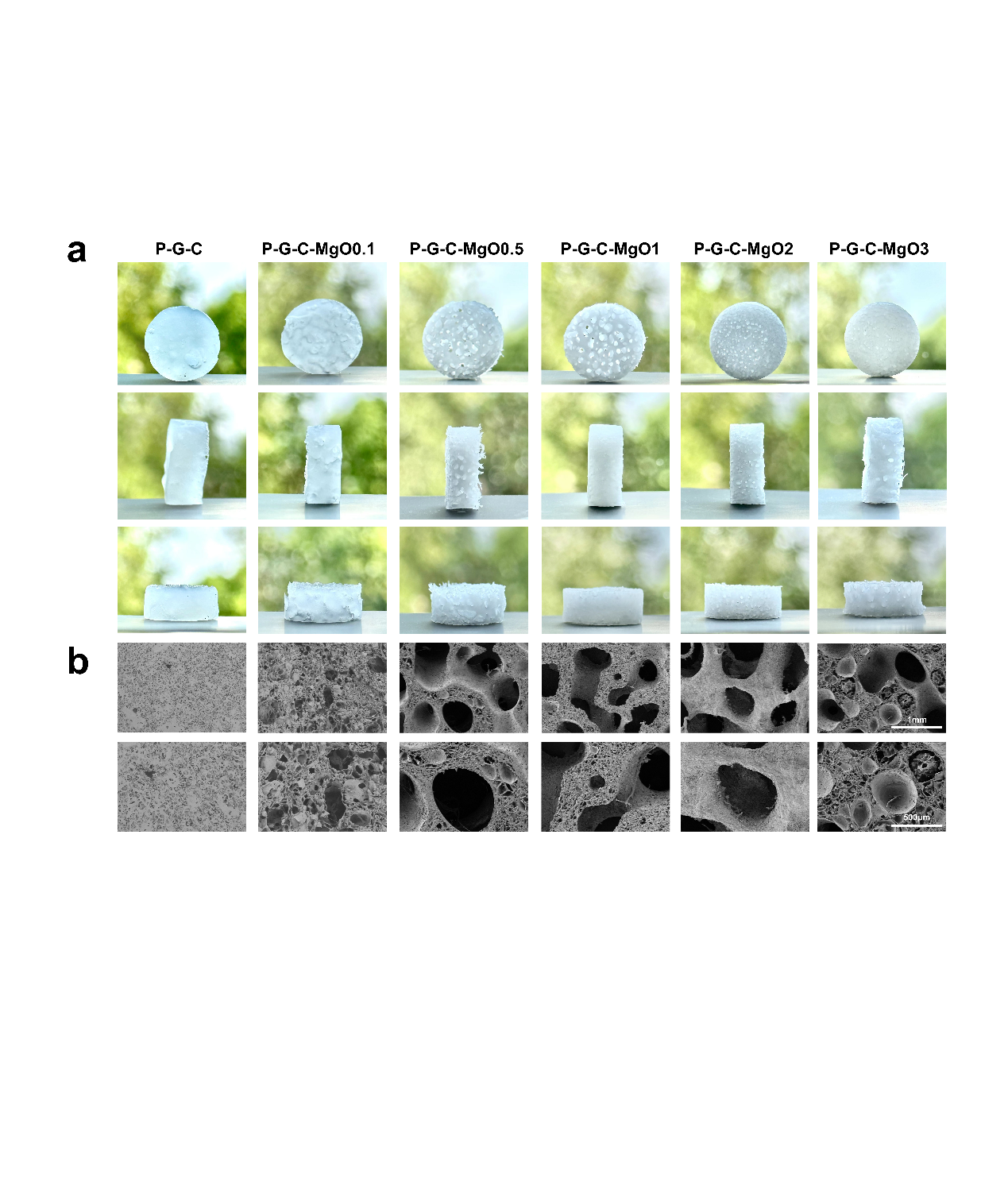
**

**Fig.S2.** **Optical photographs and SEM images of hydrogel scaffolds after *in vitro* mineralization**

**
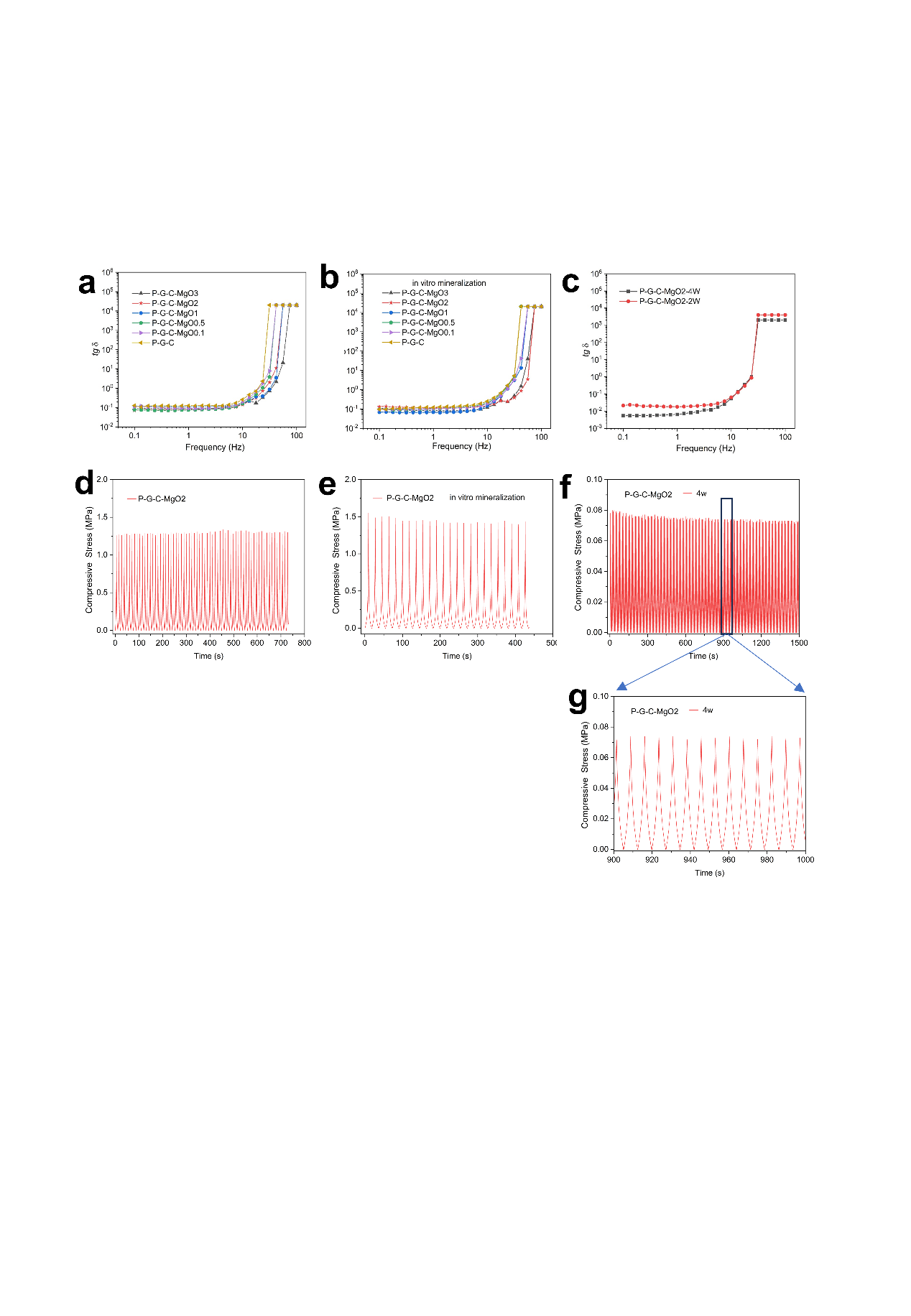
**

**Fig.S3. (a) The loss factor of each group hydrogel scaffolds prior to *in vitro* mineralization. (b) The loss factor of each group hydrogel scaffolds after *in vitro* mineralization. (c) The loss factor of P-G-C-MgO2 after 2W, 4W degradation. (d) The compressive strength modulus of P-G-C-MgO2 under cyclic compression. (e) The compressive strength modulus of P-G-C-MgO2 after in vitro mineralization under cyclic compression. (f-g) The compressive strength modulus of P-G-C-MgO2 after 4W degradation under cyclic compression.**

**
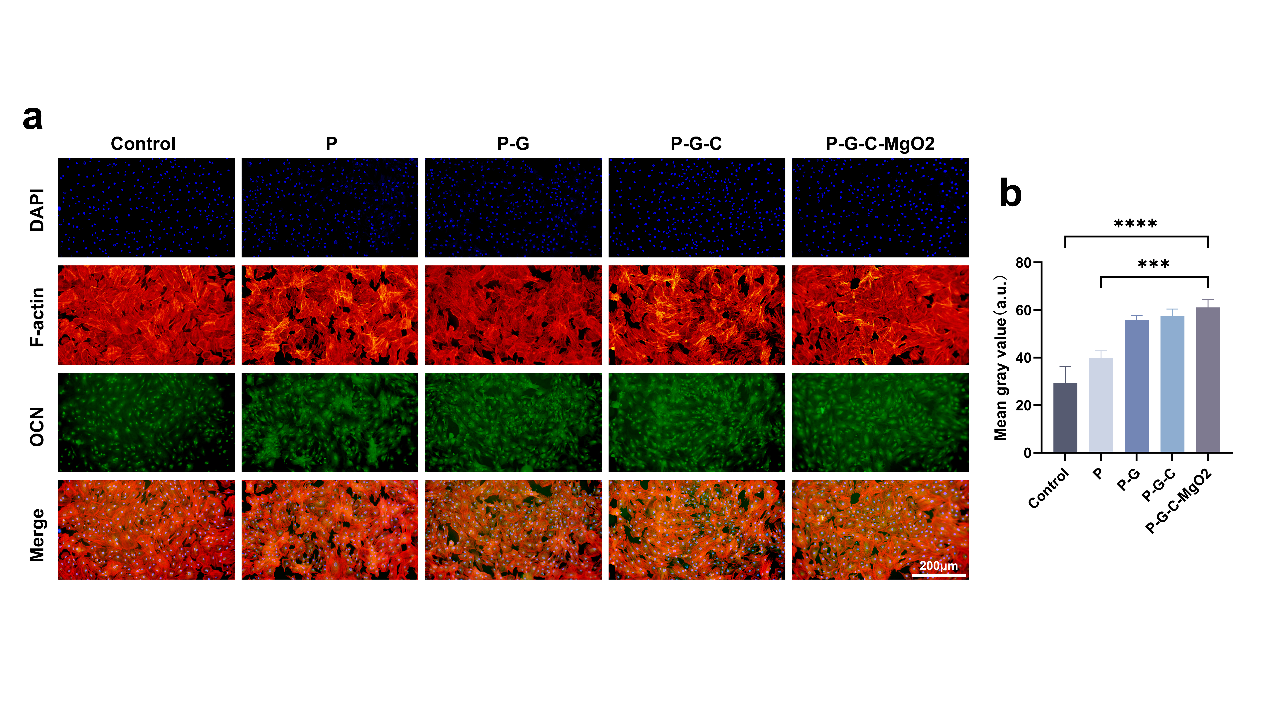
**

**Fig.S4. (a) Immunofluorescence staining of OCN for rBMSCs in different groups. (b) Quantitative analysis of the expression of OCN based on Immunofluorescence images.**

**
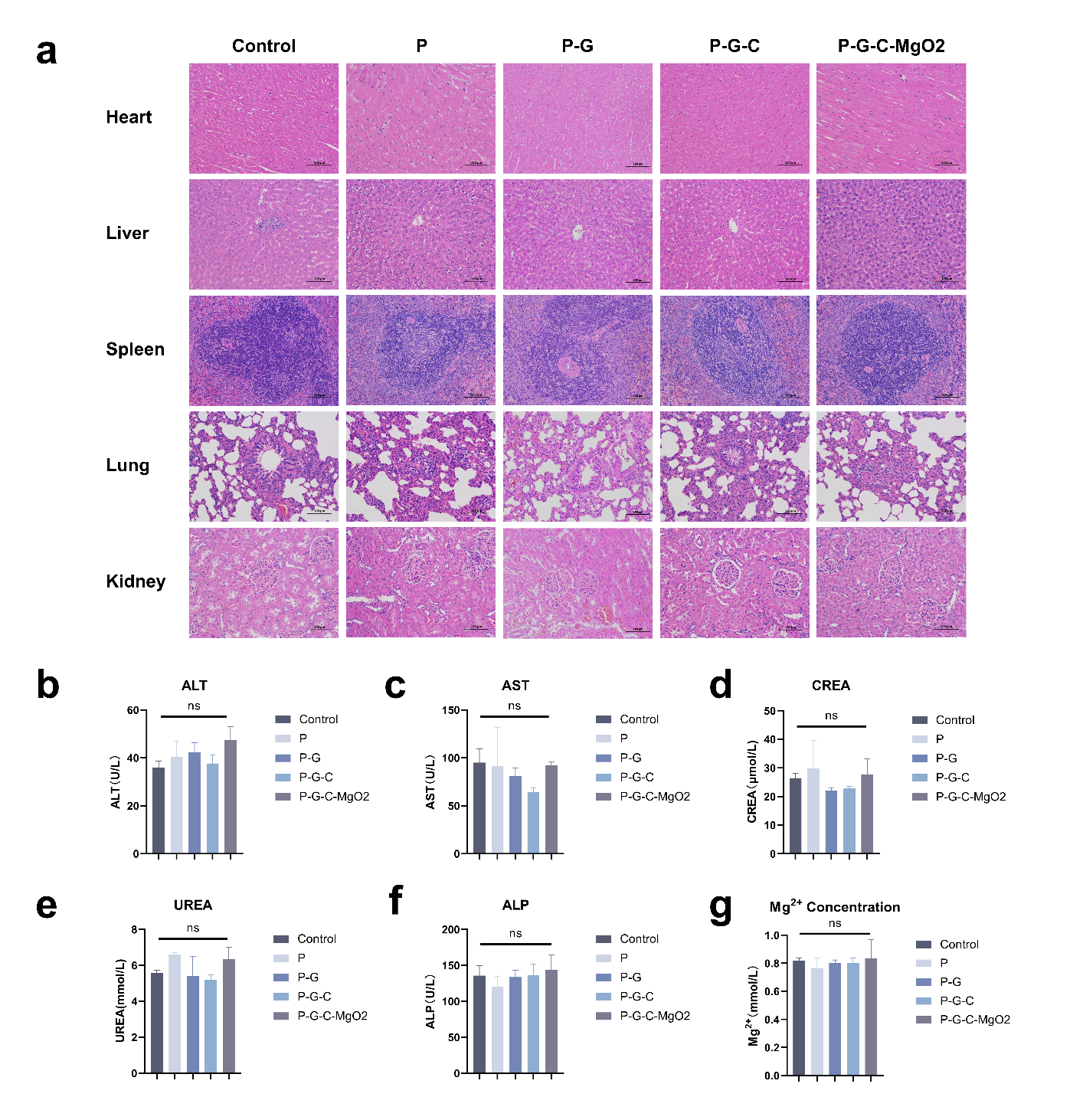
**

**Fig.S5. The H&E** **staining of organ specimens and serological indicators in each group**

**Table S1.** The primer sequences used for real-time PCR

| Gene | Forward primer | Reverse primer |
| --- | --- | --- |
| Alpl | 5^，^-GGATCAAAGCAGCATCTTACCAG-3^，^ | 5^，^-GCTTTCCCATCTTCCGACACT-3^，^ |
| SPP1 | 5^，^-GATGAACAGTATCCCGATGCCA-3^，^ | 5^，^-GTCTTCCCGTTGCTGTCCTGA-3^，^ |
| SP7 | 5^，^-GCCTACTTACCCGTCTGACTTT-3^，^ | 5^，^-GCCCACTATTGCCAACTGC-3^，^ |
| Bmp2 | 5^，^-ACCGTGCTCAGCTTCCATCAC-3^，^ | 5^，^-TTCCTGCATTTGTTCCCGAAA-3^，^ |
| β-actin | 5^，^-TGCTATGTTGCCCTAGACTTCG-3^，^ | 5^，^-GTTGGCATAGAGGTCTTTACGG-3^，^ |
